# Trends in utilisation of ultrasound by older Australians (2010–2019)

**DOI:** 10.1186/s12877-023-03771-y

**Published:** 2023-01-27

**Authors:** Virginie Gaget, Maria C. Inacio, David R. Tivey, Robert N. Jorissen, Wendy J. Babidge, Renuka Visvanathan, Guy J. Maddern

**Affiliations:** 1 Surgical Specialities, University of Adelaide, The Queen Elizabeth Hospital, Woodville, South Australia 5011 Australia; 2 Registry of Senior Australians, South Australian Health and Medical Research Institute, Adelaide, SA 5001 Australia; 3 UniSA Allied Health and Human Movement, University of South Australia, Adelaide, Australia; 4 Royal Australasian College of Surgeons, Adelaide, SA 5001 Australia; 5 Adelaide Geriatrics Training and Research With Aged Care Centre (GTRAC), Faculty of Health and Medical Sciences, University of Adelaide, Woodville, SA 5011 Australia; 6 Aged & Extended Care Services, The Queen Elizabeth Hospital, Central Adelaide Local Health Network, Woodville, SA 5011 Australia

**Keywords:** Ultrasound, Echography, Geriatrics, Epidemiology, Diagnostic imaging

## Abstract

**Background:**

Older people have increasingly complex healthcare needs, often requiring appropriate access to diagnostic imaging, an essential component of their health and disease management planning. Ultrasound is a safe imaging tool used to diagnose several conditions commonly experienced by older people such as deep vein thrombosis.

**Purpose:**

To evaluate the utilisation of major ultrasound services by Australians ≥ 65 years old between 2009- and 2019.

**Methods:**

This population-based and yearly cross-sectional study of ultrasound utilisation per 1,000 Australians ≥ 65 years old was conducted using publicly available data sources. Overall, examination site and age- and sex-specific incidence rate (IR) of ultrasound per 1,000 people, adjusted incidence rate ratios (IRRs) and 95% confidence intervals (CIs) were calculated using negative binomial regression models.

**Results:**

Over the study period, the crude utilisation of ultrasound increased by 83% in older Australians. Most ultrasound examinations were conducted on extremities (39%) and the chest (21%), with 25% of all ultrasounds investigating the vascular system. More men than women use ultrasounds of the chest (184/1,000 vs 268/1,000 people), particularly echocardiograms (177/1,000 vs 261/1,000 people), and abdomen (88/1,000 vs 92/1,000 people), especially in those ≥ 85 years old. Hip and pelvic ultrasound were used more by women than men (212/1,000 vs 182/1,000 people). There were increases in vascular abdominal (IRR:1.07, 95%CI:1.06–1.08) and extremeties (IRR:1.06, 95%CI:1.05–1.07) ultrasounds over the study period, particularly in ≥ 75 years old men.

**Conclusions:**

Ultrasound is a common and increasingly used diagnostic tool for conditions commonly experienced by older Australians.

**Supplementary Information:**

The online version contains supplementary material available at 10.1186/s12877-023-03771-y.

## Introduction

Part of the essential care required for older people is the timely and appropriate delivery of diagnostic examinations, including diagnostic imaging. In Australia in 2005, 25% of diagnostic imaging encounters were attributed to older people (≥ 65 years old) despite them representing 13% of the overall population, with X-ray being the most utilised diagnostic imaging method followed by ultrasound [1, 2]. Ultrasound is a safe imaging tool, which can be utilised in a number of settings as the equipment is easily transportable (i.e., in hospital, emergency departments, ambulance, mobile clinics) [3–6]. In the general population, ultrasound is commonly used to diagnose issues related to abdominal pain, pregnancies and reproductive organs [2]. In older people ultrasound is useful to investigate abdominal pain, liver masses, other upper abdominal mass, pancreatitis and renal issues [6, 7]. Recently, we reported a 63% national increase in the utilisation of plain X-rays between 2010 and 2019 in the general population and a 12% increase in residents of residential aged care facilities between 2009 and 2016 [8, 9]. We also reported significant national variation in the use of these services, which highlighted the lack of consistent access to diagnostic imaging and the growing need for its availability for older Australians [8–10]. A similar evaluation of the utilisation and access to ultrasound by older Australians has not been conducted.

Our study aimed to assess the utilisation of ultrasound by older Australians by 1) estimating the total usage of ultrasound services related to the examination of the chest, the abdomen and extremities, 2) identifying trends in ultrasound utilisation between 2009–10 and 2018–19, and 3) highlighting differences in service usage between age-sex groups.

## Materials and methods

### Study design, data sources and study population

We used publicly available data from 2009–10 to 2018–19 from the Medicare statistics website and the Australian Bureau of Statistics (ABS) to conduct a population based epidemiological and yearly cross-sectional study [11, 12]. All results are provided by Australian financial year from 2009–10 to 2018–19 (financial years in Australia start on the 1^st^ of July of a given year and end on the 30st of June the following year). The study population corresponds to Australians aged 65 years old or older during the study period.

### Variables

The present analysis includes ultrasound services covered by the Australian Government Medicare Benefits Schedule (MBS), which subsidises health services to citizens and permanent residents. This system covers, at least partially, diagnostic imaging evaluations ordered by a range of medical practitioners (e.g., general practitioners and specialists) [13–15]. MBS codes (i.e., codes used to claim specific services for reimbursement through Medicare) associated with ultrasound of the chest, the abdomen (including in part the urinary tract) and extremities were identified using yearly MBS listings from 2010 to 2019 (Supplementary Table 1). Codes and body parts of interest were selected due to their likely correlation with health issues commonly experienced by older citizens and for their potential to be conducted in all patients (i.e., including outpatients).

### Statistical analysis

Ultrasound services were grouped by examination site (i.e., chest, abdomen, extremities, and hip joint). Overall, age and sex specific crude and adjusted utilisation rates (incidence rate) and 95% confidence intervals (CIs) of ultrasound per 1,000 people were calculated. Changes in utilisation over time were evaluated with overall and age- and sex-adjusted incidence rate ratios (IRRs) and 95%CIs, estimated using negative binomial regression (to accommodate overdispersion in the data). A p value of < 0.05 was considered statistically significant. Statistics analyses were performed using R version 4.0.3 [16].

## Results

### Study population and total usage of ultrasound

**Fig. 1:**
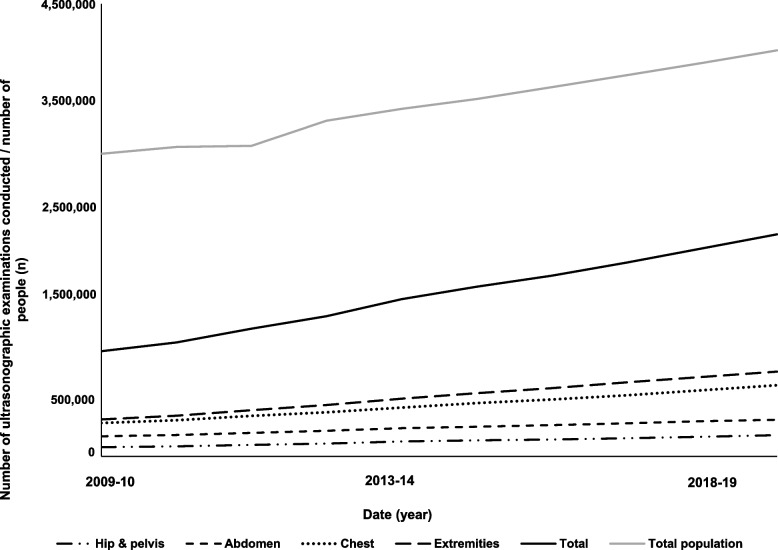
Trends in population growth and crude utilisation of ultrasound of the hip and pelvis, the abdomen, the chest and extremities between 2009–10 and 2018–19

The Australian population aged ≥ 65 years old increased from 2,914,336 in 2009–10 to 4,038,179 people in 2018–19 (Fig. 1, Supplementary Table 2). In this population, ultrasound was commonly conducted on extremities (39% in 2018–19) and the chest (21% in 2018–19) (Fig. 1). A total of 3,217,585 ultrasound examinations were conducted in 2009–10 compared to 5,900,818 in 2018–19 (Fig. 1), corresponding to an 83% increase in overall crude service (increase by examination site: chest: 116%, abdomen: 82%, extremities: 107%, pelvis and hip: 105%).

### Changes in utilisation rate of ultrasound of the chest, abodmen and extremities over time and differences across age-sex groups

**Fig. 2:**
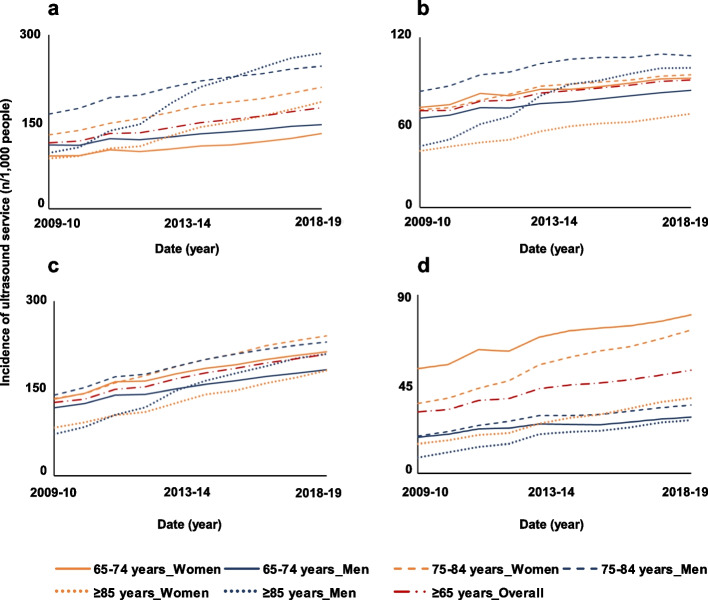
Trends in adjusted utilisation of ultrasound by older Australians for the chest (**a**), the abdomen (**b**), extremities (**c**), and the hip and pelvis (**d**)

In 2009–10, the overall utilisation of chest ultrasound was 110 services/1,000 people. This increased to 175/1,000 people in 2018–19 (Fig. 2a), with ultrasound of the heart constituting 86% of chest examinations in 2018–19. The utilisation of chest ultrasound increased over the study period (IRR:1.07, 95%CI:1.06–1.08) and was accompanied by an increase in utilisation of ultrasound of the heart (IRR:1.06, 95%CI:1.05–1.07) and of other thoracic ultrasounds (e.g., chest and abdominal wall) (IRR:1.15, 95%CI:1.14–1.16) (Table 1). The utilisation of chest ultrasound was higher in men than in women aged between 75 and 84 years old (246/1,000 vs 129/1,000) and ≥ 85 years old (268/1,000 vs 184 /1,000) (Fig. 2a).

**Table 1:** Adjusted incidence rate ratio of change in radiology utilisation by the older Australian population between 2009–10 and 2018–19, overall and by age-sex group

	Overall	Females			Males		
	≥ 65	65–74	75–84	≥ 85	65–74	75–84	≥ 85
**Ultrasound**							
Total chest	1.06 (1.06–1.07)	1.04 (1.03–1.04)	1.06 (1.05–1.06)	1.09 (1.08–1.10)	1.03 (1.03–1.04)	1.05 (1.04–1.05)	1.13 (1.11–1.15)
Heart	1.06 (1.05–1.07)	1.03 (1.03–1.03)	1.05 (1.05–1.06)	1.09 (1.08–1.10)	1.03 (1.03–1.03)	1.04 (1.04–1.05)	1.13 (1.11–1.15)
Other thorax	1.15 (1.13–1.16)	1.16 (1.13–1.18)	1.17 (1.15–1.20)	1.18 (1.16–1.20)	1.10 (1.09–1.11)	1.10 (1.10–1.11)	1.15 (1.14–1.17)
Total Abdomen	1.04 (1.04–1.05)	1.03 (1.02–1.03)	1.04 (1.03–1.04)	1.05 (1.05–1.06)	1.03 (1.02–1.03)	1.03 (1.02–1.04)	1.10 (1.08–1.12)
Abdominal vessels	1.07 (1.06–1.08)	1.05 (1.05–1.06)	1.06 (1.06–1.07)	1.10 (1.09–1.11)	1.04 (1.03–1.04)	1.05 (1.04–1.06)	1.13 (1.11–1.15)
Other abdominal	1.03 (1.02–1.04)	1.02 (1.01–1.02)	1.03 (1.02–1.03)	1.03 (1.03–1.04)	1.02 (1.02–1.03)	1.02 (1.01–1.02)	1.07 (1.05–1.09)
Total extremities	1.07 (1.06–1.08)	1.05 (1.05–1.06)	1.07 (1.06–1.08)	1.09 (1.08–1.10)	1.05 (1.04–1.05)	1.06 (1.05–1.06)	1.13 (1.11–1.14)
Upper extremities	1.08 (1.07–1.09)	1.05 (1.05–1.06)	1.08 (1.07–1.09)	1.11 (1.10–1.12)	1.05 (1.05–1.06)	1.07 (1.06–1.08)	1.14 (1.12–1.16)
Lower extremities	1.13 (1.12–1.14)	1.10 (1.09–1.11)	1.13 (1.11–1.15)	1.16 (1.14–1.17)	1.10 (1.09–1.11)	1.12 (1.10–1.13)	1.19 (1.17–1.22)
Extremities vessels	1.06 (1.05–1.06)	1.03 (1.02–1.03)	1.05 (1.05–1.06)	1.07 (1.07–1.08)	1.03 (1.02–1.03)	1.04 (1.04–1.05)	1.11 (1.10–1.13)
Total hip joint	1.08 (1.07–1.09)	1.04 (1.04–1.05)	1.08 (1.07–1.10)	1.11 (1.10–1.12)	1.04 (1.03–1.05)	1.06 (1.05–1.08)	1.13 (1.10–1.15)

All estimates were adjusted by age and gender. 95% confidence intervals are given in parentheses

The overall utilisation rate in abdominal ultrasound increased from 66/1,000 people ≥ 65 years old in 2009–10 to 90/1,000 people in 2018–19 (Fig. 2b). The increase in utilisation was statistically significant in most age-sex groups (overall: IRR:1.05, 95%CI:1.04–1.05). In people ≥ 75 years old, trends in abdominal ultrasound utilisation rate were driven by items related to the examination of abdominal vessels, which accounted for 54% of all abdominal ultrasound conducted in 2018–19. The utilisation of ultrasound of abdominal vessels by people ≥ 65 years old increased over the study period (IRR:1.07, 95%CI:1.06–1.08) (Table 1). The utilisation rate of abdominal ultrasound in people aged ≥ 75 years old was higher in men (92/1,000) compared to women (88/1,000) (Fig. 2b). The change in utilisation of abdominal ultrasound was higher in people ≥ 85 years old, with a statistically significant increase observed in men (IRR:1.10, 95%CI:1.08–1.12) and women (IRR:1.06, 95%CI:1.05–1.06).

The crude utilisation of ultrasound of extremities was of 121/1,000 people ≥ 65 years old in 2009–10 and 208/1,000 people in 2018–19 (Fig. 2c). The overall utilisation of ultrasound conducted on extremities increased over the study period (IRR:1.07, 95%CI:1.07–1.08) (Fig. 2c, Table 1). These trends were driven by items related to the ultrasound of veins and arteries in extremities, which accounted for at least 40% (range: 40%-68%) of all ultrasound examinations conducted on extremities in 2018–19 across all age-sex groups. There was a high increase in utilisation of ultrasound on extremities in people ≥ 85 years old (women: IRR:1.09, 95%CI:1.08–1.10; men: IRR:1.13, 95%CI:1.11–1.15) (Table 1). Women (212,1,000) received ultrasound of extremities more than men (182/1,000) in 2018–19.

The overall utilisation of ultrasound of the total hip joint (i.e., pelvis or hip) was of 30/1,000 people ≥ 65 years old in 2009–10 compared to 49/1,000 people in 2018–19, with a statistically significant increase (IRR:1.08, 95%CI:1.07–1.09) (Fig. 2d, Table 1). The utilisation was higher in women compared to men aged between 65 and 74 years old (79/1,000 vs28/1,000) and between 75 and 84 years old (72/1,000 vs 34 /1,000) (Fig. 2d).

### Changes in utilisation rate of heart ultrasound and differences across age-sex groups

**Fig. 3:**
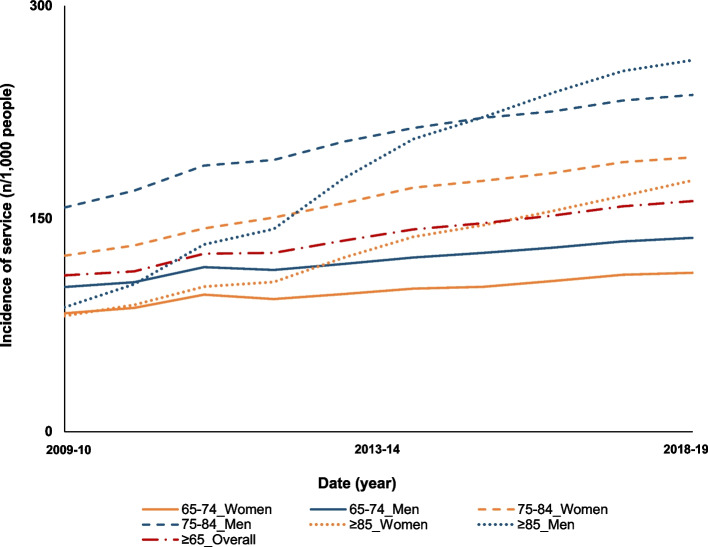
Trends in cardiac ultrasound utilisation by older Australians between 2009–10 and 2018–19

The overall utilisation of heart ultrasound was of 106/1,000 people ≥ 65 years old in 2009–10 compared to 162/1,000 people in 2018–19, with a statistically significant increase over the study period (IRR:1.06, 95%CI:1.05–1.07). In each age-sex group, heart ultrasound usage was higher in men compared to women (Fig. 3, Table 1). Heart ultrasound utilisation increased by 213% in men ≥ 85 years old compared to other age-sex groups, with 87/1,000 men in 2009–10 and 261/1,000 men in 2019 (IRR: 1.13, 95%CI:1.11–1.15) compared to 81/1,000 women in 2009–10 and 177/1,000 women in 2018–19 (Fig. 3, Table 1).

### Changes in the utilisation rate of ultrasound of vessels in the abdomen and the extremities and differences across age-sex groups

**Fig. 4:**
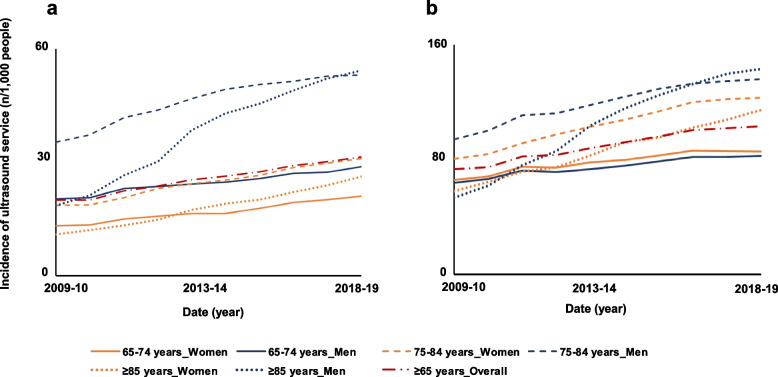
Trends in adjusted utilisation of ultrasound by older Australians between 2009–10 and 2018–19 for vessels in the abdomen (**a**) and the extremities (**b**)

The overall utilisation of ultrasound of abdominal vessels was 19/1,000 people ≥ 65 years old in 2009–10 and 32/1,000 people ≥ 65 years old in 2018–19, with a statistically significant yearly increase over the study period (IRR:1.07, 95%CI:1.06–1.08) (Table 1). The utilisation of this service was higher in men than in women, especially in those aged 65–74 years old (53/1,000 vs 31/1,000) and ≥ 85 years old (54/1,000 vs 27 USs/1,000) in 2018–19 (Fig. 4a). The utilisation of these items rose significantly for all age-sex groups, with higher yearly increases in people ≥ 85 years old (men: IRR:1.13, 95%CI:1.11–1.15; women: IRR:1.10, 95%CI:1.10–1.11).

The overall utilisation of ultrasound of vessels in extremities was of 70/1,000 people ≥ 65 years old in 2009–10 and 103/1,000 people ≥ 65 years old in 2018–19, with a statistically significant yearly increase over the study period (IRR:1.06, 95%CI:1.05–1.07) (Table 1). The occurence of this service was similar in men (105/1,000) and women (101/1,000) aged ≥ 65 years old in 2018–19 (Fig. 4b). The utilisation of these services increased yearly in the older population, especially for people aged ≥ 85 years old (men: IRR:1.12, 95%CI:1.10–1.14; women: IRR:1.08, 95%CI:1.07–1.08).

## Discussion

Over the study period, crude ultrasound ultilisation by older Australians increased by 83%, while the corresponding population increased by 38%. Similar trends were observed in Canada and the USA, with ultrasound utilisation by older people doubling between 2000 and 2016 [17]. The crude increase in ultrasound utilisation observed in Australia is higher than for plain X-ray utilisation (63%) [8]. In 2005 plain x-rays were the most claimed diagnostic imaging tool with 4.5 examinations/100 GP encounters followed by ultrasound with 2.7 examinations/100 GP encounters [2]. By 2019, the number of ultrasound procedures carried out on older Australians was almost double that of plain X-rays (*n* = 5,900,818 and 3,058,677, respectively), demonstrating considerable uptake of this diagnostic method [8].

The increase in ultrasound utilisation between 2009 and 2019 in Australia was driven by the number of ultrasound examinations for the extremities and chest. This contrasts with prior studies reporting that, amongst conditions relevant to older Australians (i.e. omission of pregnancy and fertility-related ultrasound), the leading indication for ultrasound examination in the general population was abdominal pain (5.5% of all GP encounters), followed by sprains/strains (3.5%), shoulder syndrome (3.0%), breast mass investigation (2.9%), cholecystitis (2.8%), bursitis (2.6%) and phlebitis (2.5%) [2]. The present study is therefore the first to fully capture the pattern and trends of recent ultrasound needs of older Australians.

Time trends in ultrasound utilisation varied by examination site, with a doubling in the sex-adjusted utilisation of chest ultrasound over the observation period for men ≥ 85 years old. These trends underpinned the utilisation of ultrasound of the heart, which is exacerbated in older men (i.e. ≥ 85 years old). These trends are consistent with the substantial increase in transthoracic and transoesophagal heart ultrasound between 2002 and 2013 for the overall Australian population [18]. Fonseca et al. reported that increase in utilisation varied by geographic location and did not appear to be illness-related, demonstrating the under- or over-utilisation of these two items depending on the area the patients were treated. Transthoracic and heart ultrasound remain the gold standard methods to investigate chronic heart failure, pericardial effusion, haemothorax in traumatic elderly patients and non-traumatic pleural effusions [7, 19, 20]. It is therefore possible that over-utilisation and/or changes in policies in the management of chronic heart disease may have contributed to the increase in utilisation observed during the study period. More thorough ongoing cardiac monitoring may also contribute to these figures, which is exemplified by an increase in cardiac disease survival in older Australians (-5.1% death per year in Australians ≥ 70 years old) [21].

The utilisation of abdominal ultrasound increased in most age-sex groups examined between 2009–10 and 2018–19 and was underpinned by the increase in ultrasound of abdominal vessels, which represented 54% of this class of ultrasound services. In the overall population, abdominal pain remains the main indication for ordering an abdominal ultrasound, both in public and private settings [2, 22, 23]. In 2001, a study by the Australian Institute of Health and Welfare reported that ultrasound represented 67.5% of all imaging ordered for the investigation and management of abdominal pain, followed by plain X-ray (10.1%), with patients being then referred either to a specialist (12.8% of GP accounters), allied health (1.9%), hospital services (3.3%) or emergency departments (0.8%) [13]. In 2000, older citizens (i.e., ≥ 65 years old), represented 18.5% of all patients consulting a GP for abdominal pain, of which 61.7% were then referred to receive imaging. More recent reports indicate that computed tomography (CT) now tends to be ordered in preference to other modalities for the evaluation of abdominal issues [17, 23]. Older people are major CT users in Australia and elsewhere [17, 23, 24]. In the USA for example, 11% of all emergency department presentations for abdominal pain have CT scans,[23, 24] and its use has increased tenfold between 1997 and 2016, while the utilisation of abdominal ultrasound plateaued between 1997 and 2011 [23]. In Australia, CT utilisation has increased from 33/1,000 to 112/1,000 between 1993–4 and 2012–13 beyond the increase in population, suggesting that this increase was driven practice changes, and likely subsequent increase in accessibility, subsidisation and GP ordering.[25]. It has already been demonstrated that the use of abdominal diagnostic imaging in the community has the potential to reduce the number of patients referred to a specialist and emergency department presentations, in turn offering the possibility to fast-track appropriate patient care [26–28]. Conversely, drivers non-related to illness, such as GPs’ fear of liability for missed diagnoses, can lead to over-utilisation of diagnostic imaging, and to increased health care costs [29]. Concurrently, there was a clear increase in utilisation of abdominal vascular ultrasound in the older population, and particularly in older men (i.e., ≥ 75 years old). Abdominal vascular ultrasound can be ordered to evaluate the possibility of abdominal aorta aneurysm, and particularly the presence of thrombus [19]. This condition is more common in elderly men than in women (ratio of 7:1) and is usually diagnosed by physical examination of the abdomen and ultrasound [19, 30]. The specific investigation of abdominal aorta aneurysm, could potentially explain the difference in utilisation of abdominal vascular ultrasound between men and women in the older population.

The utilisation of ultrasound of the extremities and hip joint increased during the study period, especially in older women. This increase was driven by an increase in ultrasound of veins and arteries of the extremities. In the general Australian population, ultrasound of extremities are mainly used to investigate injuries related to sprains/strains (3.5% of all GP encounters), followed by shoulder syndrome (3%), bursitis/tendonitis (2.6%) and phlebitis/thrombophlebitis (2.5%) [2]. Ultrasound, in particular compression ultrasound (B-mode imaging), is the method of choice for the diagnosis of deep vein thrombosis (DVT); a common vascular condition experienced by older people (57% of DVT cases) [31–34]. The risk for DVT increases 1% yearly in people ≥ 60 years old and is associated with serious consequences, including premature death in the older population [31, 35, 36]. Additionally, an Australian report demonstrated that ultrasound (doppler) represents 80% of all tests ordered by GPs for the investigation of peripheral vascular disease, followed by diagnostic radiology (13.3%) and CT (4.4%) [13]. These reports demonstrate that ultrasound remains the method of choice to investigate vascular issues commonly experienced by older citizens, which can partially explain the trends observed in the present study.

Our study relied on publicly available data and therefore limited our ability to examine the appropriateness of service access, the indication for services, and other important clinical characteristics that may influence the utilisation of these services [15]. Our study focused on Australian Government Medicare-subsidised services and did not capture services that may have been paid for privately, subsidised by private health insurance or special concession status (e.g. Department of Veteran’s Affairs’ card holders), or performed in state funded public hospitals, therefore likely underestimated the service utilisation rate. However, we estimate that a significant proportion of the services obtained by older Australians are captured since diagnostic imaging is used widely (39% of Australians accessed a diagnostic imaging service outside hospital settings in 2018–19 under Medicare, representing 25.7 million services). Further, it is estimated that 60% of all imaging tests are ordered by GPs in Australia, with the remainder being order mainly by specialists, which are all claimable through MBS [13, 14]. Additionally, with an adequate referral most of the services are subsidised by Medicare and therefore there is no financial incentive for individuals to access them in other ways. While this is a limitation of our study, we believe that examining the government subsidised services accessed by older Australians is still of significant interest and we do not expect this under ascertainment in the number of procedures to be differential between groups examined or overtime. The study strengths include the examination of the full Australian population and Medicare Benefits Schedule subsidised ultrasound services. Further, this study addresses specific knowledge gaps by evaluating ultrasound utilisation by age-sex groups and by examination site.

## Conclusion

The crude utilisation of ultrasound by older Australians has increased by 83% between 2009–10 and 2018–19. The changes in ultrasound utilisation observed during the study period, varied by age group, sex and examination site, with ultrasound of the chest and extremities being the most common in older people. Vascular ultrasound represented a major part of all services conducted on the abdomen and the extremities, being a leading cause for the increase. These results demonstrate that ultrasound is increasingly used to address the diagnostic needs of older Australians.

## Supplementary Information

**Additional file 1:Table S1.** MBS codes investigated.

**Additional file 2:Supplementary Table 2.** Number of Australians ≥65 years old between 2010 and 2019 by age-sex group as provided by the Australian Bureau of Statistics.

## Data Availability

All data used herein was obtained from publicly available sources and relevant data providers have been acknowledged in the text, namely the Medicare statistics website and the Australian Bureau of Statistics (ABS) [11, 12]. The access to the data is opened to everyone and no permission needs to be granted. Medicare statistics website: http://medicarestatistics.humanservices.gov.au/statistics/mbs_item.jsp Australian Bureau of Statistics website: https://www.abs.gov.au/statistics#people

## Abbreviations

ABS: Australian bureau of statistics
CI: Confidence interval
CT: Computed tomography
GI: Gastro-intestinal
GP: General practitioner
IR: Incidence rate
IRR: Incidence rate ratio
MBS: Medicare benefit schedule
