# Supplementary material for: Trends in utilisation of ultrasound by older Australians (2010–2019)
Source: BMC Geriatr. 2023 Jan 27;23:50. doi: 10.1186/s12877-023-03771-y (PMC9883967; doi:10.1186/s12877-023-03771-y)
Supplement: Supplementary file 1 — Additional file 1:Table S1. MBS codes investigated. [file 12877_2023_3771_MOESM1_ESM.pdf]

Table S1 MBS codes investigated

	Body area	MBS items
Ultrasonography		
Chest	Heart	55113, 55114, 55118, 55125
	Chest, abdominal wall	55812, 55813[&], 55814, 55125
Abdomen	Abdomen (including urinary tract)	55036, 55037, 55038, 55039
	Abdominal vessels	55276, 55278
Extremities	Hand, wrist, elbow, humerus, forearm, shoulder, upper arm	55800, 55801[&], 55802, 55803[&], 55804, 55805[&], 55806, 55807, 55808, 55809[&], 55810, 55811[&]
	Buttock, thigh, knee, lower leg, ankle, foot	55824, 55825[&], 55826, 55827, 55828, 55829[&], 55830, 55831[&], 55832, 55833[&], 55834, 55835&, 55836, 55837[&], 55838, 55839[&], 55840, 55841[&], 55842, 55843[&]
	Extremities vessels	55238, 552444, 55248, 55252
Hip and Pelvis	Hip	55816, 55817[&], 55818, 55819[&]
	Pelvis and pelvic	55044, 55045, 55065, 55067, 55068, 55069, 55731, 55733

Abbreviation: MBS: Medicare Benefit Schedule.

Note: [&] Service started after the financial year 2009/2010.
