# Supplementary material for: Trends in utilisation of ultrasound by older Australians (2010–2019)
Source: BMC Geriatr. 2023 Jan 27;23:50. doi: 10.1186/s12877-023-03771-y (PMC9883967; doi:10.1186/s12877-023-03771-y)
Supplement: Supplementary file 2 — Additional file 2:Supplementary Table 2. Number of Australians ≥65 years old between 2010 and 2019 by age-sex group as provided by the Australian Bureau of Statistics. [file 12877_2023_3771_MOESM2_ESM.pdf]

Supplementary Table 2: Number of Australians ≥65 years old between 2010 and 2019 by age-sex group as provided by the Australian Bureau of Statistics.

	2010	2011	2012	2013	2014	2015	2016	2017	2018	2019
**Men**										
65-74	761,288	822,878	813,549	920,511	956,825	989,503	1,025,951	1,057,029	1,089,381	1,114,146
75-84	439,404	448,527	448,983	470,988	485,668	495,902	510,657	531,605	552,182	579,485
≥85	127,528	138,934	139,070	156,490	164,903	171,882	179,238	185,066	190,647	198,110
Total ≥65	1,328,220	1,410,339	1,419,602	1,547,989	1,607,396	1,657,287	1,715,846	1,773,700	1,832,210	1,891,741
**Women**										
65-74	793,853	848,781	850,382	942,751	982,490	1,017,187	1,057,318	1,094,545	1,134,663	1,168,531
75-84	544,632	553,046	553,390	564,323	574,672	582,767	595,646	614,740	633,686	660,313
≥85	247,631	264,373	264,537	283,105	291,630	297,063	303,441	307,857	312,416	317,594
Total ≥65	1,586,116	1,666,200	1,668,309	1,790,179	1,848,792	1,897,017	1,956,405	2,017,142	2,080,765	2,146,438
**Persons**										
65-74	1,555,141	1,671,659	1,681,931	1,863,262	1,939,315	2,006,690	2,083,269	2,251,574	2,224,044	2,282,677
75-84	984,036	1,001,573	1,002,373	1,035,311	1,060,340	1,078,669	1,106,303	1,146,345	1,185,868	1,239,798
≥85	375,159	403,307	403,607	439,595	456,533	468,945	482,679	492,923	503,063	515,704
Total ≥65	2,914,336	3,076,539	3,087,911	3,338,168	3,456,188	3,554,304	3,790,842	3,790,842	3,912,975	4,038,179

Source: The Australian Bureau of Statistics (https://www.abs.gov.au/statistics/people/population).
